# The biomechanical effect of different posterior tibial slopes on the tibiofemoral joint after posterior-stabilized total knee arthroplasty

**DOI:** 10.1186/s13018-020-01851-y

**Published:** 2020-08-12

**Authors:** Yingpeng Wang, Songhua Yan, Jizhou Zeng, Kuan Zhang

**Affiliations:** 1 School of Biomedical Engineering, Capital Medical University, No.10 Xitoutiao, You An Men Wai, Beijing, 100069 China; 2 Beijing Key Laboratory of Fundamental Research on Biomechanics in Clinical Application, Capital Medical University, Beijing, 100069 China; 3 Department of Orthopedics, Beijing Luhe Hospital, Capital Medical University, No. 82 Xinhua South Road, Tongzhou District, Beijing, 110149 China

**Keywords:** Posterior-stabilized total knee arthroplasty, Posterior tibial slope, Posterior femoral translation, Femoral rotation, Contact area, Contact pressure

## Abstract

**Background:**

Different posterior tibial slopes (PTS) after posterior-stabilized total knee arthroplasty (PS-TKA) may lead to different biomechanical characteristics of knee joint. This cadaveric study was designed to investigate the tibiofemoral kinematics and contact pressures after PS-TKA with different PTS.

**Methods:**

Nine human cadaveric knee specimens were used for PS-TKA with the PTS of 3°, 6°, and 9°. The tibiofemoral kinematics and contact pressures were measured during knee flexion angle changing from 0 to 120° (with an increment of 10°) with an axial load of 1000 N at each angle.

**Results:**

The root mean square (RMS) of the tibiofemoral contact area and the mean and peak contact pressures during knee flexion were 586.2 mm^2^, 1.85 MPa, and 5.39 MPa before TKA and changed to 130.2 mm^2^, 7.56 MPa, and 17.98 MPa after TKA, respectively. Larger contact area and smaller mean and peak contact pressures were found in the joints with the larger PTS after TKA. The RMS differences of femoral rotation before and after TKA were more than 9.9°. The posterior translation of the lateral condyle with larger PTS was more than that with smaller PTS, while overall, the RMS differences before and after TKA were more than 11.4 mm.

**Conclusion:**

After TKA, the tibiofemoral contact area is reduced, and the contact pressure is increased greatly. Approximately 80% of the femoral rotation is lost, and only about 60% of the femoral translation of lateral condyle is recovered. TKA with larger PTS results in more posterior femoral translation, larger contact area, and smaller contact pressure, indicating that with caution, it may be beneficial to properly increase PTS for PS-TKA.

## Introduction

Total knee arthroplasty (TKA) is an effective procedure for the treatment of advanced knee joint diseases, which is aiming for restoring the function of knee joint and improving the quality of life of patients [1, 2]. However, approximately one in five patients is not satisfied with the result of the operation [3], and one of the main reasons is that knee function cannot be fully recovered after TKA [3–6]. Bourne et al. found that patient satisfaction regarding function during the performance of daily living activities varied between 70 and 84% [3]. Wylde et al. reported that only 52.2% of patients were very satisfied with returning to the normal activities of daily living, and only 43.7% patients were very satisfied with their ability to perform leisure activities [4]. The study by Wright et al. showed that, after TKA, restoration of the unimpaired functional ability is rare, with only 33% of people reporting no functional limitations with their replaced knee [5].

Kinematic parameters and contact pressures are the important factors affecting knee function after TKA. The more natural kinematics of the knee, such as femoral external rotation and posterior translation during knee flexion, result in better functional outcome and satisfaction for the patient post-TKA [6, 7]. The altered load-bearing pattern could be reflected by contact area and contact pressures that are related to aseptic loosening, which is the most common cause of surgical failure and prosthesis revision [8–10]. Stress shielding caused by the changed load-bearing patterns can subsequently result in bone remodeling, which is one of the main causes of prosthesis loosening [9, 11]. Excessive contact pressure may cause more wear of the polyethylene prosthesis, resulting in more debris and wear particles, which in turn can cause a biological response resulting in bone resorption and osteolysis that are also one of the main causes of prosthesis loosening [10, 12].

Clinical scoring systems are commonly used to assess knee function and maybe influenced by the patient’s or doctor’s perception [5, 13, 14]. Performance measures and gait analysis are also widely used to assess postoperative knee function, but only for overall motor function, not for movements and contact pressures within the knee joint [5, 13, 14]. Finite element model simulation has been carried out to calculate knee kinematics and contact pressures, but the validity of finite element model has always been questioned [10, 15, 16]. The in vivo measurement of knee contact pressure is not feasible due to practical or ethical reasons, so in vitro biomechanical experiments are often chosen to evaluate knee contact pressure. A number of studies have been carried out to successfully measure the knee kinematics and contact pressures in cadavers in vitro experiments [12, 17–23]. The advantage of in vitro experiments is the ability to examine different surgical procedures, such as osteotomy at different angles, which is difficult to achieve in in vivo experiments.

Posterior tibial slope (PTS) after proximal tibial resection is considered to be one of the important factors affecting knee kinematics following TKA [2, 13, 23–28]. Studies have shown that increasing PTS is conducive to increase the posterior femoral translation, flexion gap, and maximum flexion angle in cruciate-retaining TKA [23–26]. The finite element models have been developed to estimate the knee contact pressures with different PTS, but there is a paucity of in vitro studies evaluating the effect of PTS on the tibiofemoral joint contact pressures after posterior-stabilized (PS) TKA [10]. There are few cadaveric studies concerning the PTS in PS-TKA, and it still remains controversial whether or not increasing PTS in PS-TKA is beneficial [2, 26, 27].

Therefore, this cadaveric study was designed to investigate tibiofemoral joint kinematics and contact mechanics before and after PS-TKA and to evaluate the biomechanical effect of different PTS on the tibiofemoral joint.

## Methods

### Specimen preparation

Nine Chinese embalmed cadaveric knee specimens without significant pathological changes from 5 males (mean age, 56.2 years, range, 47~68 years) were used in this study. Those knees had little or no degenerative changes, no meniscal or ligamentous injury, and similar dimensions based on CT and MR examinations. Similar dimensions could ensure the same size prosthesis was implanted for all specimens to avoid the possible impact of different prosthesis sizes on contact pressure and kinematic results. Each knee specimen was bended between full extension and full flexion several times to ensure free flexion. The skin, subcutaneous tissue, and muscles were removed carefully while conserving the joint capsule and the imbedded collateral ligaments. The fibula was secured to the tibia bone in its anatomical position using polymethylmethacrylate (PMMA) bone cement. The femur, tibia, and fibula were cut approximately 20 cm proximal and 20 cm distal to the joint line. The proximal femur and distal tibia and fibula were potted in cylindrical molds filled with PMMA bone cement.

### TKA procedure

The nine specimens were divided into 3 groups with 3 specimens in each group to perform tibial osteotomy with PTS of 3°, 6°, or 9°. TKA was performed by an orthopedic surgeon using a PS fixed-bearing prosthesis (Chunli model XM, Beijing, China). This prosthesis has an anatomical femoral component that can effectively increase tibiofemoral contact area. The tibial component has a symmetrical design in medial and lateral condyles with a curved articular surface. The post-cam of this prosthesis has a curve-on-curve design. Medial parapatellar arthrotomy was used to expose the knee joint. The menisci and the anterior and posterior cruciate ligament were resected. The implant surgery was performed following mechanical alignment. Intramedullary alignment was used for both femur and tibia. The femoral osteotomy was performed in 6° of valgus and 3° of external rotation. Rotational alignment of the tibial component was at the medial third of tibial tuberosity. The original design of the tibial osteotomy of this prosthesis was performed with the PTS of 3~5°, while the tibial osteotomy was performed with the PTS of 3°, 6°, or 9° for the three groups respectively in this study. After osteotomy of the tibia, the PTS was measured and slightly trimmed repeatedly until the error was within 1.0°. The angle between the tibial intramedullary canal axis and the osteotomy surface was measured by an electronic protractor with two legs paralleling to the intramedullary rod and paralleling to the osteotomy surface respectively, and the intraoperative PTS was calculated by subtracting the angle from 90°. The mean PTS of the three groups of specimens after the surgeries was 2.9° (range, 2.1~3.6°), 6.2° (range, 5.6~6.9°), and 9.1° (range, 8.4~9.8°) respectively. Trial components were inserted, and ligamentous balancing was performed if the knee was judged to be tight on either the medial or the lateral side. In this study, all TKAs were performed using the same size implants. The femoral and tibial component was implanted using bone cement, and the knee joint was sutured.

### Experimental setup

**Fig. 1:**
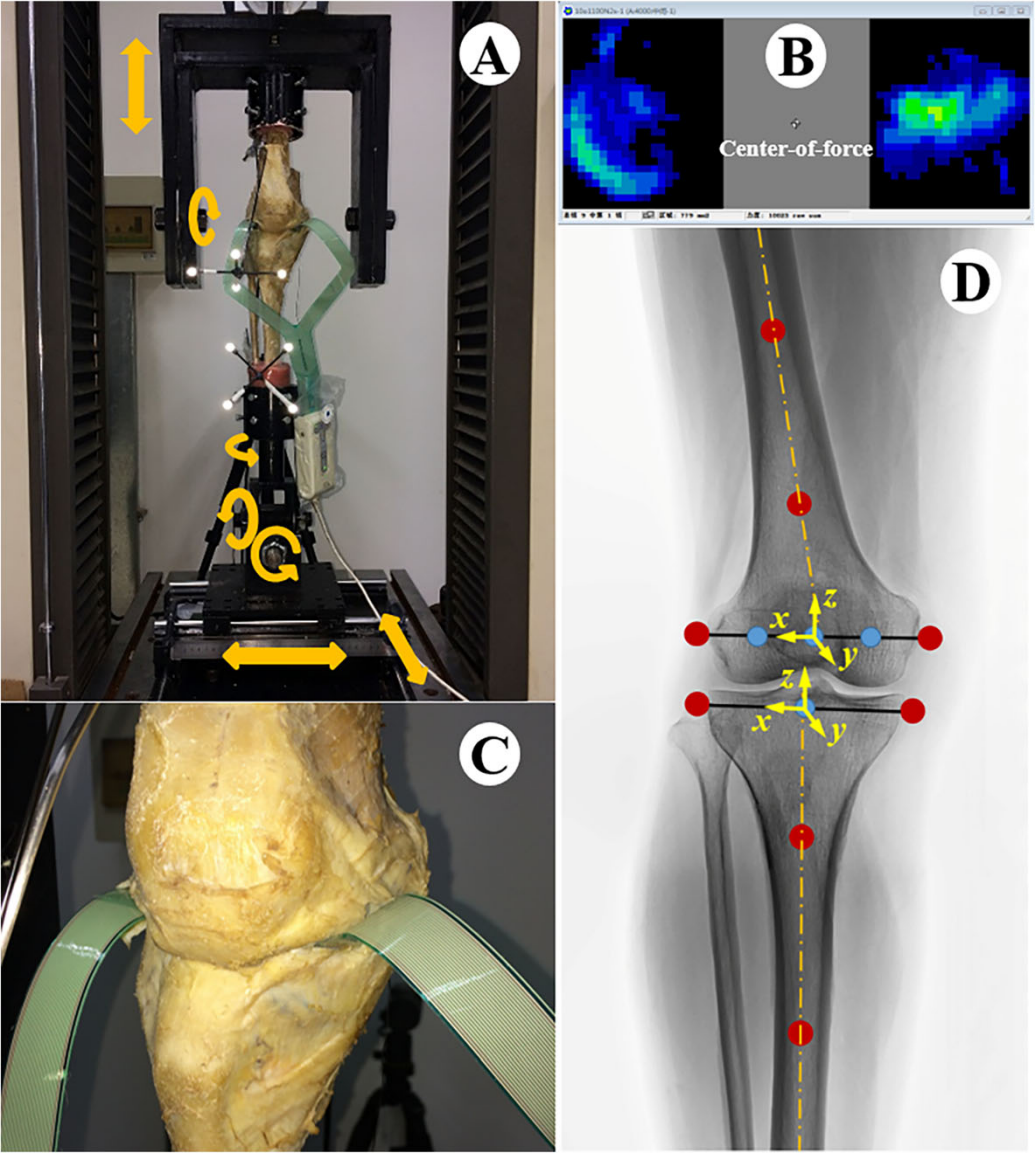
The experimental process and setup for biomechanical testing. **a** The experimental process and the custom testing jig for the femur and tibia. The arrows indicate the degrees of freedom allowed during testing (flexion/extension, internal/external rotation, varus/valgus, medial/lateral translation, anterior/posterior translation, and proximal/distal translation). **b** The real-time feedback of the contact pressure map in the Tekscan software. **c** The Tekscan sensor was inserted into the medial and lateral compartments of the tibiofemoral joint and secured to the posterior aspect of the tibia by suture anchors. **d** The local coordinate systems of the femur and tibia segments. Eight knee anatomic markers (the medial and lateral condyle of the tibia and femur, two points parallel to the shaft of the femur, and two points parallel to the shaft of the tibia) were used to create the local coordinate systems for the femur and tibia segments. The midpoint of the femoral condyle was identified as the condyle center and defined as the origin of the femur coordinate. The midpoint of the tibial condyle was defined as the origin of the tibia coordinate. The midpoints between the femoral condyle center and the medial and lateral condyle of the femur were used to represent approximately the centers of the medial and lateral condyle and to describe the posterior translations of the medial and lateral condyle

The knee was mounted in a customized testing jig that allowed or controlled 6 degrees of freedom of knee motion (Fig. 1a), in accordance with the knee biomechanical testing jig used in previous studies [17–22]. The free rotation and translations of the tibia avoided excessive tibial constraints during axial loading to ensure physiological loading during each test [22]. The jig was mounted in a materials testing machine (WDW4100, Changchun Kexin testing instruments co. LTD, China), which was used to provide axial load.

A thin (0.1 mm) pressure-sensitive sensor (Tekscan model 4000, South Boston, Massachusetts, America) was used to measure the tibiofemoral joint contact pressure divided into the lateral and medial joint compartments. The sensor of each compartment comprised a grid of 26 × 22 sensels and had was 33 × 28 mm in size overall. For each test, the contact pressure map could be recorded in real-time with the sensor, and contact area and mean and peak contact pressures could be calculated (Fig. 1b). The sensors were calibrated according to the manufacturer’s guidelines, and every sensor was used only for one specimen test. Small incisions were made on the joint capsule, and the sensor was inserted into the medial and lateral compartments of the tibiofemoral joint and secured to the posterior aspect of the tibia by suture anchors (Fig. 1c).

A three dimensional motion analysis system (Simi Motion, Simi Reality Motion Systems GmbH, Germany) with four digital cameras was used to record the kinematic data from the tibiofemoral joint. The accuracy of the system is up to 0.1 mm. Eight knee anatomic markers were used to create the local coordinate systems of the femur and the tibia segments (Fig. 1d). Two reference marker frames were rigidly fixed to the femur and the tibia to record their motion (Fig. 1a). Tibiofemoral joint kinematics were analyzed with custom MATLAB programs using the Z-Y-X Euler angle transformation.

Calculations were made of the posterior femoral translation of the lateral and medial condyle as well as the femoral rotation relative to the tibial coordinate system during knee flexion. The initial reference position was defined as the location of knee extension (flexion of 0°).

### Biomechanical testing

We performed biomechanical tests on knee joints before and after TKA. During testing, each knee specimen was first fixed at the position of full knee extension (0° flexion), and then, the knee flexion angle was manually adjusted from 0 to 120° with an increment of 10° each time. At each flexion angle (13 angles in total), 1000 N of axial compression force was applied at the knee specimen for 30 s. The load of 1000 N was consistent with the compression used in previous studies using human cadaveric knees [22]. All specimens had no damage in repeated tests. The varus-valgus alignment was also controlled to ensure that a consistent and balanced load was applied through the medial and lateral compartments throughout the testing. The center-of-force indicator from the Tekscan software output provided real-time feedback as the knee was loaded (Fig. 1b), allowing manual correction to rebalance the load on the compartments during testing. The loading was based on the assumption that the center of the force passes through the center of the knee joint. This ensured that the quantitative changes in pressure and area being measured were due solely to the changes involving the control condition and not due to changes in varus-valgus alignment. This procedure also avoided the overestimation or underestimation of the pressure in the compartments due to varus-valgus malalignment related to subtle inconsistencies in the location of the femur and tibia placement and the errors of osteotomy. No additional forces or moments were applied to the knee, and no attempt was made to place the knee in any specific position except for the flexion angle and neutral alignment. Thus, the position tested was the passive position as dictated by the knee flexion angle, neutral alignment, inherent anatomy, and applied axial load. This loading pattern has been used in numerous cadaver biomechanical studies [18–21]. The contact mechanics and kinematic data of the tibiofemoral joint were recorded with Tekscan and the Simi Motion system.

### Data analysis

We calculated the kinematic and contact mechanic parameters including the femoral external rotation, the posterior femoral translation of the condyle center, and the medial and lateral condyle, the contact area, and the mean and peak contact pressures. We also calculated the correlations and root mean square (RMS) differences between the kinematic variables before and after TKA. Correlation could reflect the degree of similarity between rotation or translation curves, and RMS differences could reflect the differences between rotation or translation ranges before and after TKA. A higher correlation coefficient with a lower RMS difference would indicate that the two kinematic patterns were similar. The RMS values throughout knee flexion were used to compare the differences in contact mechanic variables. Differences in the knee kinematic patterns and contact mechanics amongst different PTS were used to evaluate the biomechanical effects of PTS on the tibiofemoral joint.

## Results

**Fig. 2:**
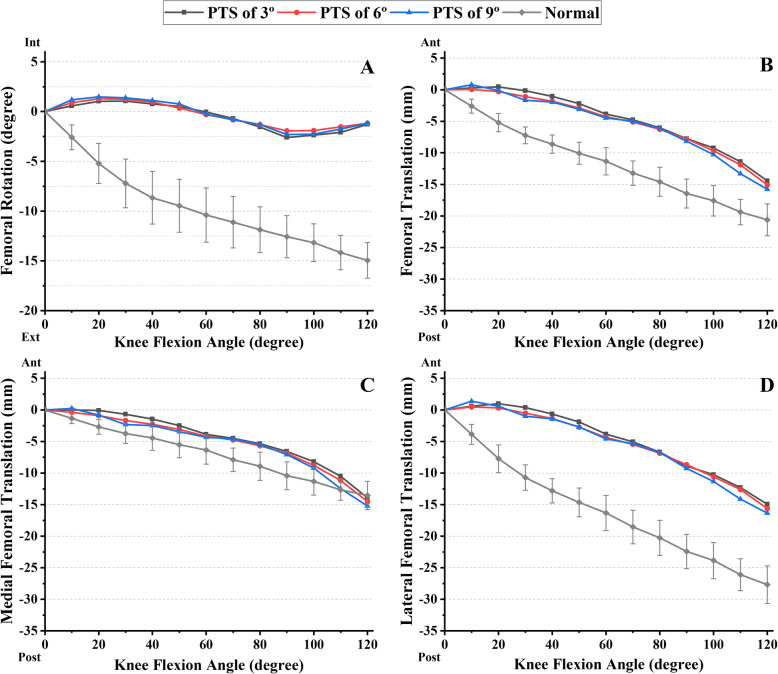
The kinematic patterns of the tibiofemoral joint in normal and TKA knees with different PTS during knee flexion. The femoral rotation relative to tibia during knee flexion is described in **a**; a positive value indicated internal rotation, and a negative value indicates external rotation. The posterior translations of the condyle center and the medial and lateral condyle relative to the tibia during knee flexion are described in **b**, **c**, and **d**, respectively; a positive value indicates anterior translation, and a negative value indicates posterior translation

Before TKA, the posterior femoral translation increased sequentially from 0 to 120° of flexion with the maximum values of 20.6 mm, 13.5 mm, and 27.7 mm for the condyle center, the medial, and lateral condyle, respectively (Fig. 2). The posterior femoral translation of the lateral condyle was greater than that of the medial condyle so that the femur exhibited gradual external rotation, reaching a maximum value of 14.9° at 120° of flexion (Fig. 2a). After TKA, the lateral femoral condyle translated slightly anteriorly before 20° of knee flexion and then posteriorly from 20 to 120° of knee flexion. The ranges of posterior translation were − 0.4~15.1 mm, 0~14.6 mm, and − 0.8~15.6 mm for the condyle center, the medial, and the lateral condyle, respectively (Fig. 2b–d). The femur rotated internally with maximum 1.2° at 20° of flexion before 60° of knee flexion and externally with maximum 2.3° at 90° of flexion after 60° of knee flexion (Fig. 2a). The posterior translation of the lateral condyle decreased dramatically after TKA, but the lateral condyle with the larger PTS translated more posteriorly than that with the smaller PTS (*r* = 0.916~0.928, RMS differences = 11.4~12.1 mm). The femoral rotation exhibited great changes following TKA with PTS of 3°, 6°, and 9° (*r* = 0.705~0.725, RMS differences = 9.9~10.1°) (Table 1).

**Table 1:** The correlations and RMS differences between the kinematic variables before and after the surgery

	PTS of 3°	PTS of 6°	PTS of 9°
FER (°)			
Correlations (*r*)	0.705	0.725	0.711
RMS differences	9.9	10.1	10.1
PFT (mm)			
Correlations (*r*)	0.935	0.942	0.941
RMS differences	7.4	6.9	6.7
M_PFT (mm)			
Correlations (*r*)	0.955	0.957	0.952
RMS differences	2.8	2.4	2.3
L_PFT (mm)			
Correlations (*r*)	0.916	0.927	0.928
RMS differences	12.1	11.7	11.4

*FER* the femoral external rotation, *PFT* the posterior femoral translation of the condyle center, *M_PFT* the posterior femoral translation of the medial condyle, *L_PFT* the posterior femoral translation of the lateral condyle

**Fig. 3:**
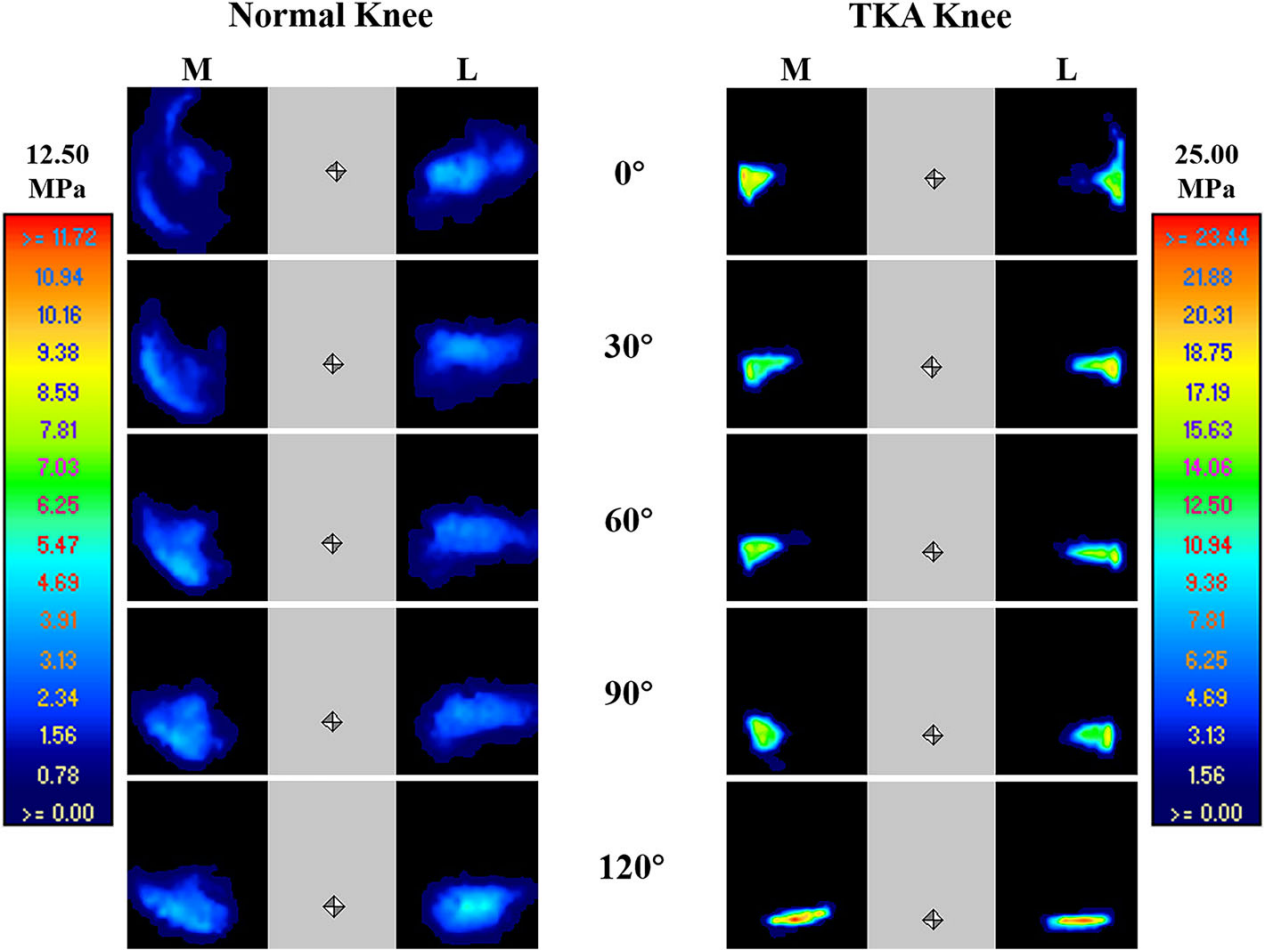
The tibiofemoral joint contact pressure map in normal and TKA knees during knee flexion

**Fig. 4:**
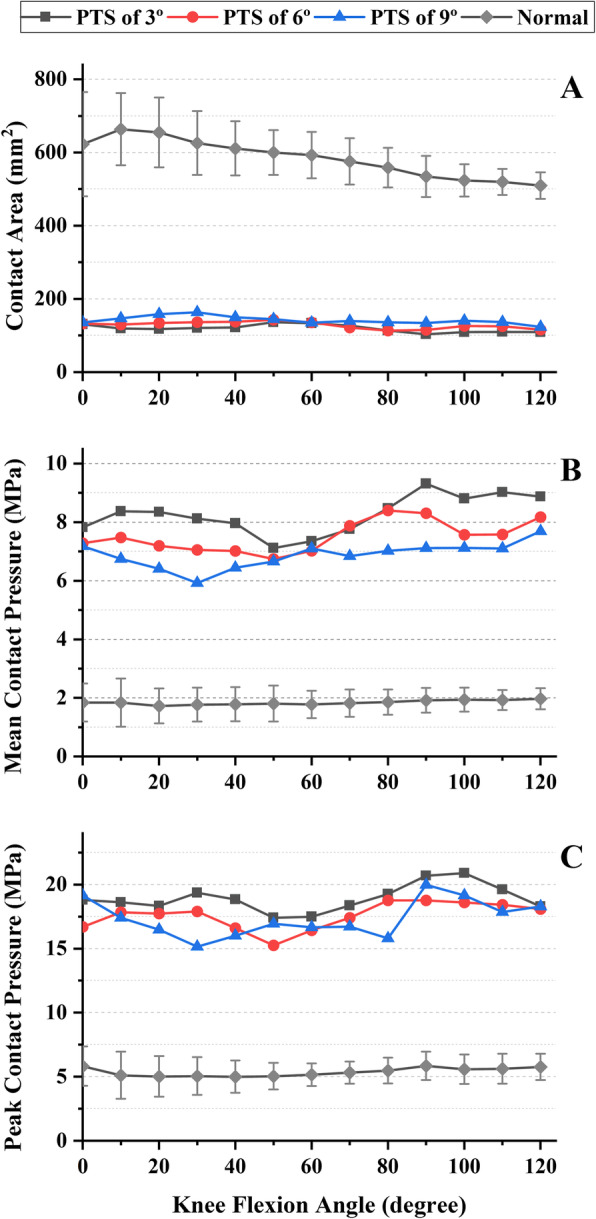
The tibiofemoral joint contact mechanics in normal and TKA knees with different PTS during knee flexion

Before TKA, the contact area was changing from 663.7 to 509.6 mm^2^ as the knee flexion angle increased. The mean and peak contact pressures were changing from 1.73 to 1.97 MPa and from 5.01 to 5.86 MPa respectively as the knee flexion angle increased (Figs. 3 and 4). After TKA, the contact area decreased significantly, varying from 103.3 to 163.7 mm^2^. The contact areas of the tibiofemoral joints fluctuated without clear patterns. The mean and peak contact pressures increased significantly, varying from 5.92 to 9.32 MPa and from 15.16 to 20.91 MPa, respectively. The contact pressure still holds the tendency to increase as the increase of knee flexion angle but with large fluctuations (Figs. 3 and 4). With the increase of PTS from 3 to 9°, the contact area RMS increased from 119.9 to 142.3, and the mean and peak contact pressure RMS decreased from 8.28 to 6.89 and 18.95 to 17.42 during knee flexion respectively (Table 2).

**Table 2:** The RMS values of postoperative contact mechanic variables throughout knee flexion

Root mean square (RMS)	PTS of 3°	PTS of 6°	PTS of 9°
Contact areas (mm^2^)			
Total compartment	119.9	128.6	142.3
Medial compartment	59.5	65.7	74.2
Lateral compartment	60.5	63.0	68.4
Mean contact pressures (MPa)			
Total compartment	8.28	7.53	6.89
Medial compartment	8.18	7.64	6.80
Lateral compartment	8.56	7.81	7.21
Peak contact pressures (MPa)			
Total compartment	18.95	17.62	17.42
Medial compartment	17.12	16.59	15.76
Lateral compartment	18.29	16.68	16.85

## Discussion

The results in the present study have shown that the tibiofemoral contact area was dramatically reduced, and the contact pressure was greatly increased after TKA. Although the femoral condyle still translated posteriorly after TKA, the range of femoral translation of the lateral condyle was significantly less than that before the surgery. The range of femoral rotation was significantly reduced, even occurring reverse rotation. The contact area increased, and the mean and peak contact pressures decreased along with increasing PTS. The differences between the posterior femoral translations before and after the surgery decreased along with the increase of PTS. Overall, the femoral external rotation was about 80% lost, and the femoral translation of the lateral condyle was only recovered about 60% after TKA, which may influence the normal activities of daily living for the patient and ultimately result in dissatisfaction with TKA.

Excessive intra-articular pressure has been recognized as an important factor in the etiology of osteoarthrosis as well as in the wear, fatigue, and failure of the ultrahigh molecular polyethylene (UHMWPE) used in total knee arthroplasty [29, 30]. Higher mean and peak contact pressures and lower contact area on the articular surface may cause greater concentration of stress and is likely to result in localized damage to the insert [12, 15, 31]. The results in this study have shown a significant decrease in the contact area, accompanied by a significant increase in the mean and peak contact pressures after TKA, with a maximum peak contact pressure of 20.91 MPa, which is close to the yield stress of approximately 22 MPa for the UHMWPE [32]. Such a dramatic change in the knee contact mechanics after TKA is caused by the great differences in bone or implant condylar surface geometry and material properties. The smaller contact area in addition to the larger and more concentrated contact pressure may increase the risk of implant damage and loosening. The results from our study have shown that with the increase of PTS, contact area became larger, and mean and peak contact pressures became smaller, which may indicate that properly increasing PTS could be beneficial to the tibiofemoral joint.

With increasing angles of knee flexion, the tibiofemoral contact area decreased gradually, and the contact pressure increased gradually. This result was consistent with a previous study that found that knee flexion reduces contact area and increases contact stresses as the smaller posterior sagittal radius of the femur articulates with the tibia [33]. Following TKA, the contact area no longer decreased gradually, and the contact pressure no longer increased gradually with knee flexion, which may be caused by the changes in the surface geometry of the artificial joint.

The posterior femoral translation has been considered as a determinant for the maximum knee flexion angle, because femoral roll back aids in the clearance of the posterior aspect of the knee, especially on the lateral side [7]. The posterior femoral translation also results in an increase of the lever arm of the quadriceps muscle, contributing to the reduced quadriceps force and patellofemoral contact force [2, 7, 28]. The normal axial rotation pattern is essential for good patellar tracking [34]. Femoral external rotation during flexion reduces the Q-angle, thus stabilizing the patella, which also reduces the patellar shear force and patellofemoral joint reaction force [7, 34]. Before TKA, the lateral femoral condyle translated more posteriorly than the medial condyle throughout knee flexion, creating a gradual external rotation of the femur with increasing knee flexion angle. But after TKA, the translation of the lateral condyle decreased significantly, and the small difference between translations of the medial and lateral condyle limited the range of femoral rotation and even caused reverse rotation. Obviously, the dramatic changes in tibiofemoral joint kinematics after TKA may cause great variation of the knee function. Therefore, achievement of better kinematic patterns will help patients in their functional performance [7]. The results from this study have demonstrated that larger PTS resulted in more posterior translation of lateral condyle than smaller PTS, indicating that properly increasing PTS could be helpful to recover posterior femoral translation of the lateral condyle after TKA, which is in agreement with previous studies [2, 16].

The findings from our study may suggest that the larger PTS following PS-TKA may lead to a positive biomechanical effect on the knee joint. However, too large PTS should be avoided because it has been shown that excessive PTS may cause anteroposterior instability leading to posterior subluxation of the tibial component, thereby increasing shear stresses on the posterior part of the tibial UHMWPE [35]. In addition, the increased PTS causes impingement of the femoral cam on the anterior aspect of the tibial post, leading to increased stress and anterior post wear and deformation [36]. An excessive increase in PTS may also lead to the progressive loosening of the tibiofemoral joint due to a reduction in collateral ligament tension and failure of the tibial post [16]. Surgeons should ensure comprehensive consideration of the various factors to determine PTS when performing TKA.

Our study had several limitations that should be considered. The knee specimens used in this study were embalmed cadavers that may underestimate the changes in contact mechanics before and after TKA. In our previous fresh-frozen specimen study, the RMS of the contact area and mean contact pressure from 0 to 30° of flexion were 860 mm^2^ and 1.23 MPa, and the peak contact pressures of the medial and lateral compartments were 4.38 MPa and 4.97 MPa [16]. In the present study, the RMS of the contact area and mean contact pressure from 0 to 30° of flexion were 642 mm^2^ and 1.79 MPa, and the peak contact pressures of the medial and lateral compartments were 4.63 MPa and 4.85 MPa. So, embalming may decrease the contact area and increase the mean contact pressure, but it has less effect on the peak contact pressure. One possible reason is the denaturation of meniscus and cartilage. Following TKA, the effect of embalming on the contact mechanics would be reduced because the meniscus and cartilage is removed and replaced with the prosthesis as we examined the postoperative contact mechanics with different PTS, rather than the differences before and after surgery. The medial condyle translated posteriorly during knee flexion and reached a maximum of 13.5 mm at 120° of flexion, and the lateral condyle translated more posteriorly than the medial condyle and reached a maximum of 27.7 mm at 120° of flexion in this study, which is very close to the results (about 15 mm and 27.5 mm) from Feng’s in vivo knee kinematics study [37]. The postoperative knee kinematics from this study is also similar to that from Steinbrück’s study in which PS-TKA was performed on fresh-frozen knee specimens [12]. The results from the study by Siston et al. also showed that there were no differences of the knee kinematics between embalmed specimens and that of fresh-frozen specimens [38]. Certainly, more studies need to carry out to confirm the similarities and differences of the biomechanical testing results between embalmed specimens and fresh specimens due to the sheer scarcity and difficulty in acquiring specimens. Like any other cadaveric study, we were unable to reproduce normal tissue biology, such as muscle contractions and healing. In addition, a uniaxial compressive force was loaded without considering any rotational or translational forces and muscle forces. Although this was a consistent and reproducible loading scheme that has been used by numerous studies and allowed for reliable comparison between different osteotomy angles, it could not replicate the variable loading experienced in the knee during functional activities [18–22]. Due to the absence of the quadriceps forces, the patella is not functioning, so it is impossible to check the patella-femoral kinematics and contact mechanics, and future studies with quadriceps function are needed to detect the biomechanics of the patella-femoral joint. The repeated testing may also introduce some additional creep between the tests. Another limitation of this study is the number of specimens used. If TKA with more tibial osteotomy angles was performed on more specimens, the detailed statistical analysis could be carried out to further examine the correlations between variables from different PTS. Only the design of PS prosthesis was examined in this study. The study results from one design may not be applied to another design due to different structures and different surgery procedures. Thus, more designs of knee prosthesis should be included in the future studies.

In conclusion, TKA causes tremendous alteration of the tibiofemoral joint kinematics and contact mechanics. After TKA, the most of femoral external rotation is lost, and only partial posterior femoral translation of the lateral condyle is recovered. The contact area of the tibiofemoral joint is dramatically reduced, and the mean and peak contact pressures of the tibiofemoral joint are increased greatly. TKA with larger PTS results in more posterior femoral translation, larger contact area, and smaller contact pressure, indicating that it may be beneficial to properly increase PTS in PS-TKA with caution.

## Data Availability

The dataset supporting the conclusions of this article is included within the article. All data are fully available without restriction.

## Abbreviations

TKA: Total knee arthroplasty
PTS: Posterior tibial slope
PS: Posterior-stabilized
RMS: Root mean square
UHMWPE: Ultrahigh molecular polyethylene
